# Objective cognitive performance and subjective complaints in patients with chronic Q fever or Q fever fatigue syndrome

**DOI:** 10.1186/s12879-020-05118-z

**Published:** 2020-06-05

**Authors:** Daphne F. M. Reukers, Justine Aaronson, Joris A. F. van Loenhout, Birte Meyering, Koos van der Velden, Jeannine L. A. Hautvast, Cornelia H. M. van Jaarsveld, Roy P. C. Kessels

**Affiliations:** 1 Department of Primary and Community Care, Radboud Institute for Health Sciences, Radboud University Medical Center, PO Box 9101, 6500 HB Nijmegen, The Netherlands; 2 Department of Medical Psychology, Donders Center for Medical Neuroscience, Radboud University Medical Center, Nijmegen, the Netherlands; 3 Centre for Research on the Epidemiology of Disasters (CRED), Institute of Health and Society, Université Catholique de Louvain, Brussels, Belgium; 4 Donders Institute for Brain, Cognition and Behaviour, Radboud University, Nijmegen, the Netherlands

**Keywords:** *Coxiella Burnetii*, Post-infectious fatigue syndrome, Bacterial endocarditis, Neuropsychological test, Performance validity, Cognitive symptoms

## Abstract

**Background:**

Primary aim of this study was to compare cognitive performance of patients with chronic Q fever or Q fever fatigue syndrome (QFS) to matched controls from the general population, while taking performance validity into account. Second, we investigated whether objective cognitive performance was related to subjective cognitive complaints or psychological wellbeing.

**Methods:**

Cognitive functioning was assessed with a neuropsychological test battery measuring the domains of processing speed, episodic memory, working memory and executive functioning. Tests for performance validity and premorbid intelligence were also included. Validated questionnaires were administered to assess self-reported fatigue, depressive symptoms and cognitive complaints.

**Results:**

In total, 30 patients with chronic Q fever, 32 with QFS and 35 controls were included. A high percentage of chronic Q fever patients showed poor performance validity (38%) compared to controls (14%, *p* = 0.066). After exclusion of participants showing poor performance validity, no significant differences between patients and controls were found in the cognitive domains. QFS patients reported a high level of cognitive complaints compared to controls (41.2 vs 30.4, *p* = 0.023). Cognitive complaints were not significantly related to cognitive performance in any of the domains for this patient group.

**Conclusions:**

The high level of self-reported cognitive complaints in QFS patients does not indicate cognitive impairment. A large proportion of the chronic Q fever patients showed suboptimal mental effort during neuropsychological assessment. More research into the underlying explanations is needed. Our findings stress the importance of assessing cognitive functioning by neuropsychological examination including performance validity, rather than only measuring subjective cognitive complaints.

## Background

Q fever is an infectious disease caused by the bacterium *Coxiella burnetii.* Humans infected with *C. Burnettii* remain asymptomatic in about 60% of the cases [1]. The other 40% of infected individuals develop symptoms, ranging from a mild flu-like and usually self-limiting disease to more serious conditions such as pericarditis or myocarditis [2]. Approximately 1–5% of Q fever patients develop a chronic infection, commonly manifested as endocarditis or vascular infection, called chronic Q fever [1]. Chronic Q fever occurs primarily in patients with pre-existing cardiac valvulopathies, vascular abnormalities, or immunosuppression [2]. Another long-term manifestation of Q fever is called Q fever fatigue syndrome (QFS) [3], which consists of severe debilitating fatigue lasting for more than 6 months, experienced by patients months or years after the acute Q fever infection [4, 5].

Previous studies suggest that Q fever infection may affect cognitive functioning. A study by van Loenhout et al. showed that 20% of working Q fever patients reported concentration and memory complaints 12 months after the onset of the infection [6]. Moreover, Cvejic et al. examined changes in cognitive performance over time in a group of Q fever patients and found that processing and response speed on complex or high-attention-demanding tasks was significantly reduced during the symptomatic phase of Q fever, compared to the phase of complete recovery [7]. To our knowledge, the extent to which cognitive deficits are present in patients with chronic Q fever or QFS in the long term has not been studied yet.

Studies have found that symptoms including fatigue, pain and emotional distress, as well as possible secondary gain (e.g., disability benefits) may affect the amount of mental effort that patients undergoing neuropsychological assessment display [8, 9]. As a result, patients may adjust their efforts in order to correspond with personal expectations on cognitive performance, either intentionally or unintentionally [10, 11]. This can lead to invalid neuropsychological test performances. Performance validity tests can be used to detect suboptimal mental effort.

The aim of this study was to explore the impact of chronic Q fever and QFS on cognitive performance of patients in comparison to the general population and normative data. Performance validity assessment was included to take suboptimal mental effort into account. In addition, we aimed to assess the relation between subjective cognitive complaints and objective cognitive performance. This study will add to the understanding of the clinical implications of chronic Q fever and QFS patients and will therefore provide clinicians with evidence to help formulate guidelines for the recovery and treatment of these patients.

## Methods

### Participants

Chronic Q fever patients were recruited through physicians from hospitals that were located in the region with the highest number of acute Q fever infections during the epidemic (Radboud University Medical Center Nijmegen; Jeroen Bosch Hospital, ‘s-Hertogenbosch; Bernhoven Hospital, Uden; St. Elisabeth Hospital, Tilburg, the Netherlands). All patients who met the criteria of proven or probable chronic Q fever, according to the Dutch consensus guidelines on chronic Q fever diagnostics were eligible for participation in this study [12, 13] (Appendix Table 1). QFS patients were invited through their attending physician of the Radboud university medical center, which is the QFS expert center in the Netherlands [14]. QFS patients diagnosed after the onset of the Q fever epidemic in the Netherlands of 2007, according to the Dutch multidisciplinary guideline concerning fatigue after Q fever infection [3, 5] were eligible for this study (Appendix Table 1). Patients were invited for participation independent of their disease status, that is, unrelated to whether they were still under treatment at the time of inclusion. Controls were recruited from the general population trough advertisements in a local newspaper. Controls were excluded if they had received a positive Q fever diagnosis at any time in their life, thereby excluding a diagnosis of chronic Q fever or QFS. Snowball sampling through participants with consent was used to include more participants in the control group. All participants (patients and controls) were initially recruited as part of a cross-sectional questionnaire study on long-term (5 to 9 years after acute Q fever infection) psychosocial functioning and work status of chronic Q fever or QFS patients (ImpaQt study, unpublished). Of these participants, 86% gave consent to be contacted for follow-up research. A subsample of these patients was selected based on age, sex and education level in order to obtain a representative sample. Two separate control groups were created that were comparable to each patient group, matched at group level on age, sex and education level as much as possible. Patients or controls reporting any medical conditions that (might) cause cognitive dysfunction (e.g., dementia, stroke or traumatic brain injury), as well as patients with a history of major psychiatric disorders, or patients aged under 18, were excluded from participation in this study.

The required sample size was calculated with an α of 0.05 and β of 0.20. A large effect size (Cohen’s d of 0.8) was used, as we were mainly interested in large and clinically relevant differences in cognitive performance between patients and controls. Based on these assumptions, sample sizes of *n* = 31 for each patient group and *n* = 23 in every matched control group were required. These sample sizes correspond with previous studies on neuropsychological functioning [15–17].

### Neuropsychological assessment

Cognitive performance was assessed by a neuropsychological test battery measuring four cognitive domains: episodic memory, working memory, processing speed and executive functioning. Episodic memory, the ability to encode, store and retrieve everyday information, was assessed by the immediate and delayed recall measures of the Dutch version of the Rey Auditory Verbal Learning Test [18]. Working Memory, the ability to temporally store and manipulate information, was measured with the Digit Span test [19]. Processing speed, the pace at which information is taken in, comprehended and responded to, often expressed in terms of response time, was assessed by the Trail Making Test Part A, Stroop Color-Word test Cards I and II and the Letter Digit Substitution test [20–22]. Executive functioning, a set of higher-order cognitive processes including cognitive inhibition, mental flexibility, planning and problem-solving, was measured with the Trail Making Test part B, Stroop Interference score and Category Fluency test [20, 21, 23]. Categorization of tests into cognitive domains was based on conventional classification as described in the standard textbook of neuropsychological assessment [24]. The test battery was administered by a trained research assistant during a home visit. The tests were administered in fixed order and took approximately 1 h.

Performance validity was assessed using the short form of the Amsterdam Short-Term Memory Test (ASTMT) [25–27]. A score lower than 82 was used as a cut-off for suboptimal effort [28], with a specificity of 97% and a sensitivity of 65%.

Premorbid IQ was estimated with the Dutch version of the National Adult Reading Test (NART-IQ) [29]. Fatigue was assessed using the Checklist Individual Strength (CIS-Fatigue) [30] and depressive symptoms using the Beck Depression Inventory - Short Form (BDI) [31]. These questionnaires were completed during the home visit.

The level of cognitive complaints was measured in the ImpaQt study using the Cognitive Failure Questionnaire (CFQ) [32]. On average, the questionnaire was completed 6 months before the cognitive testing, with a range of 2–9 months.

### Data analysis

Data were entered into a secured electronic case report form (Castor EDC) and analysed with IBM SPSS Statistics version 22. Each patient group was compared to their corresponding control group with respect to demographic and background variables (age, gender, educational level, premorbid IQ, performance validity, fatigue, depressive symptoms, cognitive complaints), using either a T-test for continuous variables or a Chi-Square test for categorical variables.

Each neuropsychological test score was transformed into standardized z-scores based on the distribution of the total sample, with higher scores indicating a better performance. Domain scores were calculated by averaging the z-scores of tests within a specific domain. The mean scores for each domain were compared between the patient group and the corresponding control group using Student’s t-tests and MANCOVA to adjust for possible differences in age and IQ between groups. MANCOVA analyses were repeated excluding participants who were classified as displaying poor performance validity.

Furthermore, the correlations between the four cognitive domains and self-reported scores on fatigue, depressive symptoms and cognitive complaints were calculated using the non-parametric Spearman’s rho in chronic Q fever patients and QFS patients, excluding participants with poor performance validity.

A normative comparison was also performed, to assess whether the cognitive performance of a participant was impaired compared to available normative data from healthy controls. Data were uploaded into the Advanced Neuropsychological Diagnostics Infrastructure (ANDI), a large database containing scores on neuropsychological tests from healthy participants in the Netherlands and Flanders (Belgium) [33, 34]. The deviation from the normative mean, adjusted for age and educational level, was computed for each test score separately. Performance of each participant on each test was rated with a score in three categories: a score of 0 was classified as “normal” (above − 1 SD from the norm), a score of 1 was classified as “below average” (between − 1 and − 1.65 SD from the norm) and a score of 2 was “impaired” (below − 1.65 SD from the norm). A cognitive domain as a whole was classified as “impaired” if the average rating of tests in that domain was “below average” (mean score of 1 or higher). If one or more cognitive domains were classified as impaired, a participant was classified as Cognitively Impaired, No Dementia (CIND) [35, 36]. Subsequently, the ANDI database also provided a multivariate normative comparison (MNC) for each participant, by calculating the difference from the norm for all test scores combined into one score (impaired or non-impaired [27]. The proportion of study participants classified as CIND or classified as impaired based on the MNC were compared between each Q fever group and their control group, excluding participants with poor performance validity, using Chi-square tests.

### Ethics

The Medical Ethical Review Board of the CMO Region Arnhem-Nijmegen reviewed and approved the study protocol (NL58482.091.16). All participants gave written informed consent. At the end of the assessment all participants received a gift card with a value of €10.

## Results

### Characteristics and self-reported symptoms of study populations

In total, 30 chronic Q fever patients, 32 QFS patients and 35 controls were recruited, of which 23 matched the chronic Q fever group and 21 matched the QFS group (9 controls were included in both matched control groups, 14 of the 23 and 12 of the 21 matched controls were unique). Table 1 shows the characteristics of the two patient groups and their matched controls. The chronic Q fever patients were significantly older (71 vs 67 years, *p* = 0.042) and QFS patients significantly younger (49 vs 57 years, *p* = 0.011) than their respective control groups. Both patient groups had a lower estimated premorbid IQ than their control groups (93.5 vs 102.9 *p* = 0.034 in chronic Q fever, 90.4 vs 97.8 *p* = 0.050 in QFS). A higher proportion of chronic Q fever patients showed poor performance validity (37.9%) than the other groups (range 14.3–21.1%). There are no significant differences in age, gender, educational level, premorbid IQ, level of fatigue or cognitive complaints between chronic Q fever patients showing good versus poor effort (*p*-values > 0.292). There was weak statistical evidence that more depressive symptoms in chronic Q fever patients corresponded with poor effort (*p* = 0.080). Both QFS and chronic Q fever patients reported higher levels of fatigue (44.7 and 30.3, respectively) than their control groups (24.5, *p* < 0.001 in QFS controls, 20.3, *p* = 0.002 in chronic Q fever controls). QFS patients also reported more cognitive complaints compared to controls (41.2 vs 30.4, *p* = 0.023).

**Table 1:** Characteristics and self-reported questionnaires in chronic Q fever patients, QFS patients and control groups

		Chronic Q fever	Control group	***p***-value	QFS	Control group	***p***-value
		(*n* = 30)	(*n* = 23)		(*n* = 32)	(*n* = 21)	
Age (in years)	mean (SD)	71 (8.2)	67 (7.4)	0.042	49 (12.6)	57 (10.5)	0.011
Gender (male)	n (%)	24 (80.0%)	17 (73.9%)	0.600	16 (50.0%)	11 (52.4%)	0.865
Educational level							
Low	n (%)	12 (40.0%)	6 (26.1%)	0.561	8 (25.0%)	7 (33.3%)	0.488
Middle	n (%)	9 (30.0%)	8 (34.8%)		16 (50.0%)	7 (33.3%)	
High	n (%)	9 (30.0%)	9 (39.1%)		8 (25.0%)	7 (33.3%)	
Premorbid IQ ^a^	mean (SD)	93.5 (16.6)	102.9 (14.4)	0.034	90.4 (11.8)	97.8 (14.5)	0.050
Poor performance validity ^b^	n (%)	11 (37.9%)	3 (14.3%)	0.066	5 (16.1%)	4 (21.1%)	0.660
Self-reported questionnaires							
Fatigue	mean (SD)	30.3 (12.1)	20.3 (9.4)	0.002	44.7 (7.6)	24.5 (13.6)	< 0.001
Depressive symptoms	mean (SD)	1.6 (2.4)	1.1 (1.9)	0.439	3.3 (3.1)	1.6 (3.2)	0.066
Cognitive complaints	mean (SD)	25.3 (10.0)	27.8 (11.6)	0.394	41.2 (17.7)	30.4 (14.2)	0.023

^a^1 missing value in QFS patients

^b^1 missing value in chronic Q fever patients, 2 missing values in both control groups, 1 missing value in QFS patients

### Cognitive performance compared to control groups

Table 2 shows that, without adjusting for age and premorbid IQ score between groups, chronic Q fever patients performed significantly worse in the domains episodic memory (*p* < 0.001), working memory (*p* = 0.018) and executive functioning (*p* = 0.006) compared to their control group. However, after adjusting for age and premorbid IQ, only the difference between chronic Q fever patients and their control group in the domain of episodic memory remained statistically significant (*p* = 0.035). Moreover, after excluding participants who displayed poor performance validity from both groups, chronic Q fever patients did not perform significantly worse in any of the domains compared to their control group. There were no significant differences between QFS patients and their controls, neither before nor after adjusting for age and premorbid IQ, or after excluding participants with poor performance validity.

**Table 2:** Comparison of cognitive domains with MANCOVA, unadjusted and adjusted for age and premorbid IQ, for all study participants and separately for participants without poor performance validity

	Chronic Q fever		Control group			QFS		Control group		
	Mean	(SE)	Mean	(SE)	*p*-value	Mean	(SE)	Mean	(SE)	*p*-value
**All participants (unadjusted)**	*n* = 30		*n* = 23			*n* = 32		*n* = 21		
Episodic Memory	−0.752	(0.160)	0.162	(0.183)	< 0.001	0.397	(0.155)	0.112	(0.192)	0.255
Working Memory	−0.330	(0.142)	0.195	(0.162)	0.018	− 0.006	(0.152)	0.337	(0.187)	0.160
Processing Speed	−0.163	(0.089)	−0.070	(0.101)	0.493	0.135	(0.065)	0.108	(0.081)	0.798
Executive Functions	−0.316	(0.116)	0.188	(0.132)	0.006	0.027	(0.110)	0.195	(0.136)	0.342
**All participants (adjusted**^**1**^**)**	*n* = 30		*n* = 23			*n* = 32		*n* = 21		
Episodic Memory	−0.595	(0.158)	−0.043	(0.183)	0.035	0.373	(0.147)	0.192	(0.181)	0.243
Working Memory	−0.232	(0.134)	0.067	(0.156)	0.175	0.056	(0.132)	0.304	(0.163)	0.218
Processing Speed	−0.080	(0.086)	−0.177	(0.099)	0.488	0.096	(0.068)	0.160	(0.084)	0.113
Executive Functions	−0.211	(0.115)	0.052	(0.134)	0.165	0.048	(0.111)	0.157	(0.137)	0.184
**Excluding participants with poor performance validity**	*n* = 18^a^		*n* = 18^b^			*n* = 25^a,c^		*n* = 15^b^		
Episodic Memory	−0.117	(0.184)	0.193	(0.184)	0.283	0.589	(0.151)	0.319	(0.199)	0.305
Working Memory	−0.134	(0.214)	0.260	(0.214)	0.241	0.188	(0.148)	0.496	(0.195)	0.234
Processing Speed	0.111	(0.093)	−0.139	(0.093)	0.091	0.080	(0.084)	0.186	(0.111)	0.467
Executive Functions	−0.016	(0.133)	0.222	(0.133)	0.253	0.192	(0.095)	0.319	(0.125)	0.440

^1^Adjusted for age and premorbid IQ

^a^1 missing value of performance validity

^b^2 missing values of performance validity

^c^1 missing value of premorbid IQ

### Relationship between cognitive performance and self-reported symptoms

Table 3 shows the correlations between the four cognitive domains and self-reported symptoms. Only the correlations between the domain of episodic memory and cognitive complaints in chronic Q fever patients (rho = 0.686, *p* = 0.002) and the correlation between episodic memory and fatigue in QFS patients (rho = 0.480, *p* = 0.013) were statistically significant. These correlations indicate that a better performance in the domain of episodic memory was related to more cognitive complaints or a higher level of fatigue.

**Table 3:** Correlations between self-reported questionnaires and cognitive domains with Spearman Rho, excluding participants with poor performance validity in chronic Q fever and QFS patients

	Fatigue		Depressive symptoms		Cognitive complaints	
	Correlation Coefficient	*P*-value	Correlation Coefficient	*P*-value	Correlation Coefficient	*P*-value
**Chronic Q fever patients (*n*** **= 18)**						
Episodic Memory	0.005	0.985	−0.120	0.636	0.686	0.002
Working Memory	0.117	0.645	0.124	0.623	0.408	0.093
Processing Speed	0.149	0.555	0.086	0.733	−0.012	0.961
Executive Functions	−0.128	0.612	0.102	0.687	0.420	0.082
**QFS patients** **(*n*** **= 25)**						
Episodic Memory	0.480	0.013	−0.194	0.342	0.214	0.294
Working Memory	0.191	0.349	−0.288	0.154	0.209	0.305
Processing Speed	0.000	0.999	−0.232	0.253	−0.140	0.495
Executive Functions	0.195	0.340	0.062	0.762	−0.120	0.558

### Cognitive performance compared to normative data

There were no participants fulfilling the CIND criteria after excluding participants with poor performance validity. Chronic Q fever patients were less often classified as impaired by the MNC (0.0%, *n* = 0) than their control group (16.7%, *n* = 3), although the difference failed to reach statistical significance (*p* = 0.07). The MNC did not result in differences in cognitive impairment between QFS patients (15.4%, *n* = 4) and their control group (6.7%, *n* = 1, *p* = 0.411).

## Discussion

To our knowledge, this is the first study examining cognitive performance in QFS and chronic Q fever patients. The results of this study show that the cognitive performance of both QFS and chronic Q fever patients is not impaired compared to either a control group from the general population or normative data in the domains of episodic memory, working memory, processing speed and executive functioning. This is in contrast to the higher levels of reported cognitive complaints in QFS patients compared to the general population. Furthermore, we observed a high percentage of poor performance validity in chronic Q fever patients (38%) compared to the other groups (14–21%).

The discrepancy between objective measurements of cognitive performance and subjective reporting of cognitive failures that was detected in QFS patients has also been reported in patients with chronic fatigue syndrome (CFS). Wearden et al. concluded that self-reported cognitive complaints of non-depressed CFS patients were unrelated to the results from objective cognitive measures [16]. Furthermore, Cockshell et al. also concluded that subjective and objective measures of cognitive functioning in CFS patients were not related [37]. Patients may underestimate their subjective cognitive performance in self-reported questionnaires, while performing within the normative range on neuropsychological tests, sometimes even resulting in counterintuitive findings (e.g. the positive correlation between better episodic memory performance and level of subjective cognitive complaints). Also, cognitive complaints might be capturing a different construct than actual cognitive performance, and a high level of complaints might be explained by other factors than a cognitive deficit [37]. Therefore, it is important to also assess cognitive performance using objective neuropsychological tests, rather than only rely on self-report questionnaires on cognitive complaints.

Several studies in CFS patients and patients with a history of Lyme disease supported our finding that objective performance on cognitive tasks was not impaired in patients in comparison to controls. Ray et al. showed that CFS patients reported more problems in attention and other cognitive domains than healthy controls. However, their performance on several objective measures, such as the Stroop Color-Word Test (measuring processing speed and executive function) and the Embedded Figures Test, was comparable to the performance of healthy controls [15]. Shadick et al. showed that, although persons with Lyme disease (mean of 6.0 years after infection) report more memory complaints than persons without a history of Lyme disease, no difference was found in cognitive performance between these two groups [38]. Several studies did show impairment on cognitive functioning in CFS patients compared to healthy controls, however, none of these studies took performance validity into account [39–43].

There are several possible explanations for the high number of chronic Q fever patients with poor performance validity, but we were unable to confirm these hypotheses in this study. First, depressive symptoms could have affected test behavior. Chronic Q fever patients showing poor effort reported a higher level of depressive symptoms than patients showing good effort, although not statistically significant. The ‘Q fever claim’ that was ongoing during the data collection phase of this study should also be taken into account. A collective of 300 Q fever patients had started a lawsuit for compensation from the Dutch government for neglecting to inform the public about the dangers of Q fever and not taking adequate measures to protect the public from these dangers [44]. Others have also reported suboptimal cognitive performance in neuropsychological assessment especially in patients involved in ongoing litigation or compensatory claims [9, 45]. However, since QFS patients were also involved in this claim, it is unlikely that this caused a high percentage of poor performance validity in chronic Q fever patients only. Also, it should be noted that the cognitive assessment performed here was done in the context of scientific research, in which the individual test results were not reported to the participants, making it unlikely that they would ‘malinger’ for the purpose of secondary gain. Furthermore, chronic Q-fever infection can manifest itself differently between cases and is most commonly observed as either endocarditis or vascular infection [1]. More research is needed on whether these manifestations have a different impact on cognitive performance. As there is a lack of scientific literature in this field, this might also provide insights for bacterial endocarditis or vascular infection caused by other pathogens.

Our study had some limitations. Due to the high number of chronic Q fever patients with poor performance validity, the analysis could only be performed in a valid sample of 18 chronic Q fever patients. This is below the number that was necessary according to the sample size calculation and may have been insufficient to detect relevant effect sizes. However, it should be stressed that taking performance validity into account is a strength of our study design. Also, despite the attempt to match the control groups with the patient groups as much as possible, there were still slight differences in age and premorbid IQ between these two groups. Even though we statistically adjusted for these factors in our analyses, it cannot be ruled out that these differences might have had an effect on the results. These factors have both been shown to correlate highly with cognitive functioning. Therefore, the results for the chronic Q-fever patients might have been overestimated, as they were both older and had a lower premorbid IQ. For the QFS patients we do not expect that these differences have impacted our results, as this group was younger, but also had a lower premorbid IQ compared to the control group. Furthermore, the data on subjective cognitive complaints were obtained on average 6 months before the objective assessment. However, as the Q-fever related complaints were chronic in nature, we do not expect any major changes in the level of these complaints over time. Also, participants did not show any significant changes in other self-reported complaints during this time gap, such as the level of depressive symptoms.

## Conclusions

Cognitive performance of Q fever patients should be assessed with neuropsychological tests, rather than relying on subjective measures of cognitive complaints alone. In addition, this study emphasizes the need to include measures of performance validity in research on cognitive performance among Q fever patients. More research is needed to explain the high percentage of poor performance validity in chronic Q fever patients.

## Supplementary information

**Additional file 1 **: **Table 1.** Diagnostic criteria for QFS and chronic Q fever.

## Data Availability

The datasets used and/or analyzed during the current study are available from the corresponding author on reasonable request.

## Abbreviations

ANDI: Advanced Neuropsychological Diagnostics Infrastructure
ASTMT: Amsterdam Short-Term Memory Test
BDI: Beck Depression Inventory
CFQ: Cognitive Failure Questionnaire
CIND: Cognitively Impaired, No Dementia
CIS: Checklist Individual Strength
MNC: Multivariate Normative Comparison
NART-IQ: National Adult Reading Test
QFS: Q Fever Fatigue Syndrome
