# Supplementary material for: Objective cognitive performance and subjective complaints in patients with chronic Q fever or Q fever fatigue syndrome
Source: BMC Infect Dis. 2020 Jun 5;20:397. doi: 10.1186/s12879-020-05118-z (PMC7275429; doi:10.1186/s12879-020-05118-z)
Supplement: Supplementary file 1 — Additional file 1 : Table 1. Diagnostic criteria for QFS and chronic Q fever. [file 12879_2020_5118_MOESM1_ESM.docx]

# Supplementary material

| Table 1 Diagnostic criteria for QFS and chronic Q fever | | |
| --- | --- | --- |
| **QFS (5)** | **Chronic Q fever (12)** | |
|  | **Proven** | **Probable** |
| Persisting fatigue longer than six months  **AND**  Laboratory-confirmed acute Q fever, but no chronic Q fever  **AND**  No existing somatic or psychiatric co-morbidity, which could explain the fatigue  **AND**  Fatigue causes significant limitations in daily functioning  **AND**  Complaints of fatigue were not present prior to the acute Q fever infection or the complaints have since then clearly increased in severity. | 1. Positive *C. Burnetii* PCR in blood or tissue in absence of acute infection  **OR**  2. IFA ≥ 1:1024 for *C. Burnetii* phase I IgG  **AND**  - definite endocarditis according to the modified Duke criteria (45)  **OR**  - proven large vessel or prosthetic infection by imaging studies (FDG-PET, CT, MRI) | IFA ≥ 1:1024 for *C. Burnetii* phase I IgG  **AND**  One or more of the following criteria:  - Valvulopathy not meeting the major  criteria of the modified Duke criteria (45)  - Known aneurysm and/or vascular or  cardiac valve prosthesis without signs of  infection by means of TEE/TTE, FDG-PET, CT, MRI or abdominal doppler ultrasound  - Suspected osteomyelitis or hepatitis as  manifestation of chronic Q fever  - Pregnancy  - Symptoms and signs of chronic infection,  such as fever, weight loss and night  sweats, hepatosplenomegaly, persistent raised ESR and CRP  - Granulomatous tissue inflammation,  proven by histological examination  - Immunocompromised state |
| QFS: Q fever fatigue syndrome, PCR: polymerase chain reaction, IFA: immunofluorescence assay, FDG-PET: (fluorodeoxyglucose) positron emission tomography, CT: computed tomography, MRI: magnetic resonance imaging, TEE: transesophageal echocardiography, TTE: transthoracic echocardiography, ESR: erythrocyte sedimentation rate, CRP: C-reactive protein | | |
